# Cerebrospinal fluid biomarkers in Alzheimer’s disease, vascular dementia and ischemic stroke patients: a critical analysis

**DOI:** 10.1007/s00415-013-7047-3

**Published:** 2013-07-23

**Authors:** Lisa Kaerst, Andre Kuhlmann, Dirk Wedekind, Katharina Stoeck, Peter Lange, Inga Zerr

**Affiliations:** 1 Department of Neurology, Clinical Dementia Center, University Medical Center, Georg August University Göttingen, Göttingen, Germany; 2 Department of Psychiatry and Psychotherapy, Georg August University Göttingen, Göttingen, Germany

**Keywords:** CSF, Biomarkers, Aß, Tau, Alzheimer’s disease, Vascular dementia, Stroke

## Abstract

Vascular factors are thought to contribute to the development of disease pathology in neurodegenerative dementia such as Alzheimer’s disease (AD). Another entity, called vascular dementia (VaD), comprises a less defined group of dementia patients having various vascular diseases that especially emerge in the elderly population and require valid options for examination and differential diagnosis. In the context of a retrospective study, we analyzed the cerebrospinal fluid (CSF) biomarkers t-tau, p-tau and Aß42 of a total of 131 patients with AD (*n* = 47), mild cognitive impairment (MCI) (*n* = 22), VaD (*n* = 44) and stroke (*n* = 18). We found a remarkable alteration in CSF biomarker profile in AD, VaD and in acute ischemic events. CSF profile in AD patients was altered in a very similar way as in stroke patients, without statistical differences. In stroke, increase depend largely on size and duration after the initial event. Total tau levels were useful to differ between VaD and stroke. Aß42 decreased in a similar way in AD, VaD and stroke and had a trend to lower levels in MCI but not in controls.

## Introduction

Dementias due to neurodegenerative diseases are frequent in the elderly population but at the same time, vascular pathology is prevalent in the same age groups, too. Vascular factors contribute to AD pathology and perivascular Aß amyloid deposits have been observed in both AD and vascular dementia (VaD). The correct identification of patients with dementia might be hampered by the possible overlap with stroke events. An ischemic event will significantly lead into the aggravation of the clinical symptoms or might even trigger clinically yet unapparent disease, which might unmask after a strategic stroke. These interactions are not well understood and only limited information is available in the literature how CSF biomarkers which are used in dementia diagnosis might be influenced by ischemic events. If so, a careful exclusion of acute ischemic events will be necessary to support the common dementia diagnosis in elderly persons, and clinicians should take into account the possibility of a silent stroke [1]. We identified a series of patients with acute ischemic events as detected by MRI in combination with a poor performance in neuropsychological tests. In this study, we performed an analysis to see if CSF biomarkers might be altered in the same way in dementia and after stroke.

## Methods

### Patient’s data collection and analysis

Patients having different forms of neurological diseases who underwent lumbar puncture for diagnostic purposes which included CSF dementia marker profile, were analyzed. We selected data from those with a clinical diagnosis of AD (*n* = 47), mild cognitive impairment (MCI) (*n* = 22), VaD (*n* = 44) and stroke (*n* = 18, including three patients with previously diagnosed AD and a recent stroke) for further analysis (Table 1). For statistical analysis we considered all 18 patients with stroke, including three patients with AD and stroke.

**Table 1:** Clinical characteristics of patients included in the study

Diagnosis	*n*	Age in years**	Gender m:f	Duration in months***	Severity****
Control*					
Arithmetic mean	22	58.9	10:11	(*n* = 9) 84.8	
Min		47		9	
Max		72		240	
MCI					
Arithmetic mean	22	69.8	14:8	(*n* = 11) 22.5	0 = 2 1 = 19 2 = 1
Min		55		3	
Max		86		60	
AD					
Arithmetic mean	47	70.5	11:36	(*n* = 32) 25.1	0 = 1 1 = 18 2 = 20 3 = 8
Min		32		1	
Max		87		84	
VaD					
Arithmetic mean	44	73.9	21:23	(*n* = 22) 26.7	1 = 26 2 = 13 3 = 5
Min		53		2	
Max		86		108	
Stroke					
Arithmetic mean	18	72.6	10:8	(*n* = 12) 1.4	1 = 6 2 = 7 3 = 4 4 = 1
Min		51		0	
Max		86		3	

0 not determined, 1 still independent, 2 need of care, 3 high need of care, 4 exitus

* controls = patients with depression and other non-neurological diseases

** age = age at CSF analysis

*** corresponds to the period passed by since the first diagnosis of disease

**** number of patients dedicated to a certain degree of self-dependence determined by MMSE, DemTect and their symptoms

All tests were performed in the Neurochemistry Laboratory at the Department of Neurology, University Medical School, Göttingen. CSF was obtained by lumbar puncture and processed immediately. CSF was examined for standard parameters such as cell count, proteins and immunoglobulins, and tau, phosphorylated tau and amyloid-β_1–42_ according to established protocols. CSF tau protein was quantitatively analysed using a commercially available ELISA kit according to manufacturer’s instruction (INNOTEST^®^ hTAU Ag, Innogenetics). Human tau, phosphorylated at Thr181 (phosphorylated tau) was measured quantitatively with a commercially available ELISA kit [INNOTEST^®^ PHOSPHO-TAU(181P), Innogenetics]. A pathological elevated phosphorylated tau level was considered at >61 pg/ml according to manufacturer’s instruction, aimed at the diagnosis of Alzheimer’s disease. Amyloid β_1–42_ was detected with a commercially available ELISA kit [INNOTEST^®^ ß- AMYLOID(1–42) Innogenetics] for quantitative analysis. A pathological decreased amyloid β_1–42_ assay was considered at <450 pg/ml according to manufacturer’s instruction. Amyloid β_1–40_ was detected by ELISA (Genetics Company, Schlieren, Switzerland). The study was approved by the local ethics committee (16 July 2010). The diagnoses of the patients were based on the following criteria:

AD: The diagnosis was based on recent criteria ICD-10 definition for Alzheimer’s disease (F.00 G.30)
MCI: The diagnosis was based on neuropsychological evaluation (decline in MMSE)
Stroke: The diagnosis was based on clinical syndrome and neuroimaging (CT, MRI)
VaD: The diagnosis was based on ICD 10 definition (F 01)

### Statistical evaluation

The ANOVA test (Levene, Bonferroni and Tamhane T2) and Kruskal–Wallis test were used to compare the values. Values of *p* < 0.05 were considered to be significant. The BOX-Plot was used for the graph. The statistical analyses were done using IBM SPSS Statistics 19.

## Results

**Fig. 1:**
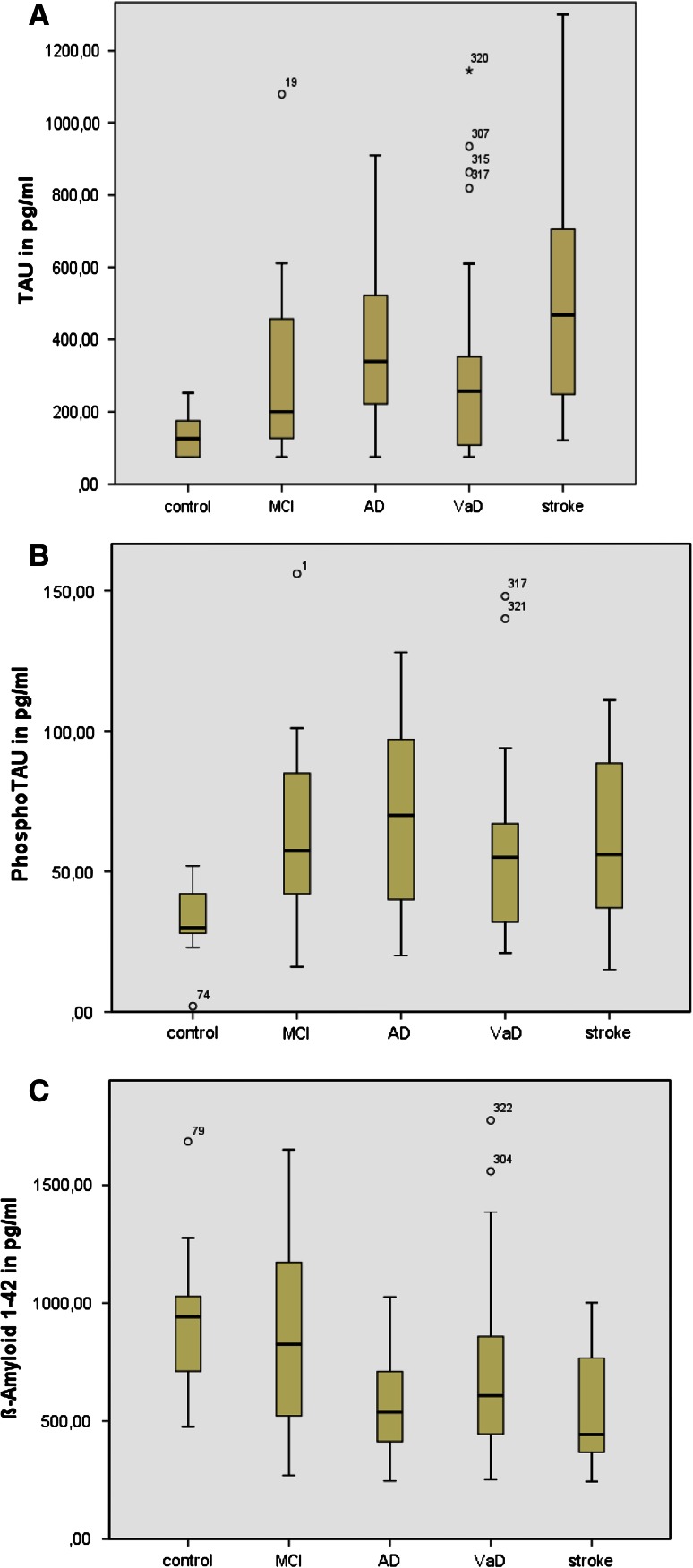
**a** [t-Tau] CSF levels across the groups **b** [p-Tau] CSF levels across the groups **c** [Aß42] CSF levels across the groups

We report similarities in the CSF profiles of those with AD and those who underwent lumbar puncture after stroke. Apart from their different sizes and the distribution of gender, both groups are especially comparable concerning age (AD arithmetic mean of 70.5 years, stroke 72.6 years) (Tables 1 and 2). Increased tau and decreased Aß42 were similar in patients with AD and in those after stroke. The highest total-tau levels were found in the stroke group (arithmetic mean 516 pg/ml, median 468 pg/ml). The highest p-tau levels were seen in AD patients, followed by MCI, stroke and VD. Also Aß40 levels were low in AD and stroke patients (Table 2; Fig. 1).

**Table 2:** Cerebrospinal fluid levels of tau, p-tau, Aß_1–42_ and Aß_1–40_ stratified by disease type

CSF	Controls*	MCI	AD	VaD**	Stroke
pg/ml	Mean (SD)	Mean (SD)	Mean (SD)	Mean (SD)	Mean (SD)
	Median	Median	Median	Median	Median
	(min–max) *n*	(min–max) *n*	(min–max) *n*	(min–max) *n*	(min–max) *n*
Tau	131 (58)	311 (243)	391 (232)	302 (252)	516 (317)
	126	200.5	339	258	468
	(75–253)	(75–1,080)	(75–910)	(75–1,145)	(121–1,300)
	21	22	47	44	18
p-tau	33 (15)	65 (35)	74 (44)	58 (34)	62 (36)
	30	57.5	70	55	56
	(2–52)	(16–156)	(20–243)	(21–148)	(15–111)
	10	14	31	21	7
Aß42	927 (164)	856 (362)	580 (211)	701 (341)	553 (245)
	942	825.5	537.5	608	443
	(476–1,685)	(256–1,650)	(246–1,026)	(251–1,775)	(243–1,002)
	21	22	46	44	17
Aß40	6,421 (2,045)	9,437 (3,081)	7,062 (1,890)	9,964 (5,639)	6,258 (2,328)
	5,885.5	9,830.5	6,843	8,276	5,846
	(4,288–11,031)	(3,321–13,729)	(3,604–11,257)	(4,493–29,786)	(3,356–9,599)
	12	12	24	20	5

* controls: patients with depression

** VaD without recent stroke

T-tau levels of AD-patients (arithmetic mean 391 pg/ml) showed less variation than in stroke (arithmetic mean 516 pg/ml). In VaD, t-tau levels were significantly lower than in stroke patients (arithmetic mean 302 pg/ml) (*p* = 0.018). There were no significant differences between stroke (arithmetic mean 62 pg/ml) and AD (arithmetic mean 74 pg/ml), *p* = 0.465, AD and MCI (arithmetic mean 65 pg/ml), *p* = 1.0 and AD and VaD (arithmetic mean 58 pg/ml), *p* = 0.579. The highest values of p-tau were found in AD, followed by MCI, similar to stroke and finally VaD (Table 2). As a biomarker, p-tau was not useful to distinguish between any of the groups, although higher levels are observed in AD.

For Aß42, there was a significant difference between AD (arithmetic mean 580 pg/ml) and MCI (arithmetic mean 856 pg/ml), *p* = 0.015, as well as between MCI and stroke (arithmetic mean 553 pg/ml), *p* = 0.022. No difference was observed for AD and stroke (*p* = 0.999), which points out the similarity between CSF Aß42 levels in both. Similar findings were obtained for Aß42 in stroke and VaD (arithmetic mean 701 pg/ml), *p* = 0.341. Aß42 levels in AD and VaD were in the same range (*p* = 0.247).

No significant differences between the groups were found when we calculated the Aß ratio [*p*-values between 0.757 and 1.0, proof for homogeneity of variances among the single groups (data not shown)].

**Fig. 2:**
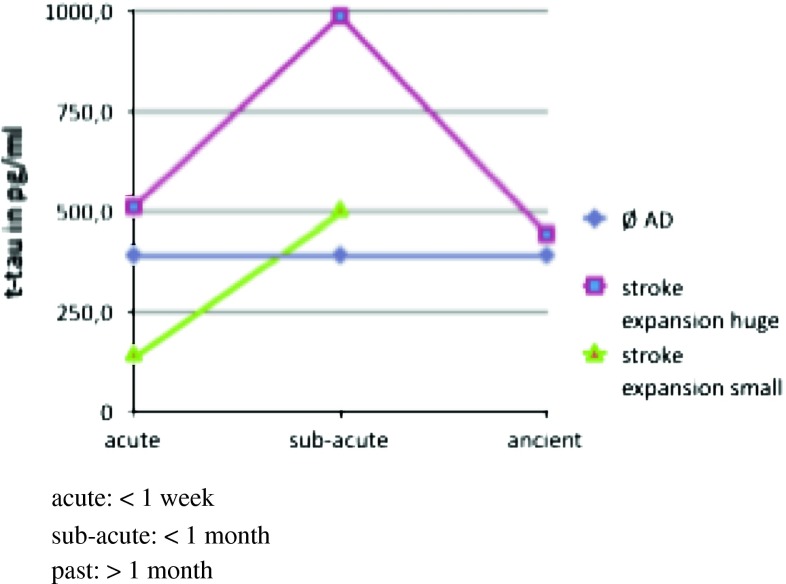
Tau-values in pg/ml after stroke defined by the size of damage and time point after the event

In this study we were specifically interested in total tau levels in patients after stroke. Because the time of the lumbar puncture varied from the day of ischemic event up to several weeks after and because the sizes and localization of the lesion generally affects the CSF protein profile (middle brain, thalamus, capsula interna and media total infarction), the tau values vary to a great extend (Fig. 2). The tau levels were even still increased within the period over 1 month, reaching values of 1,300 pg/ml in one patient.

We stratified the single patients to different subgroups considering the infarction size, time of lumbar puncture related to the time of stroke. The lowest levels were observed in stroke patients with an acute and limited brain volume damage, such as pons ischemia (*n* = 2, tau 144 and 121 pg/ml), while the highest were observed in a patient 1 month after a media infarction (tau 1,300 pg/ml). As final result, we found highest tau levels in patients with large stroke areas and subacute stage (media total infarction and multiple strokes, *n* = 3, arithmetic mean 986 pg/ml), followed by those patients with similar location but in acute stage (*n* = 4, arithmetic mean 510 pg/ml), followed by those with only limited infarction size (*n* = 2, arithmetic mean 132.5 pg/ml). Of importance, even small infarct areas led into increased CSF tau levels, especially several days after the stroke.

## Discussion

The CSF analysis offers an excellent opportunity to detect early signs of neuronal degeneration and this has been widely used for AD and other dementia disorders. However, for correct interpretation, information on potential confounding factors is extremely important. Thus, we studied CSF alterations in CSF marker profiles of commonly used AD biomarkers in patients with AD, VaD and stroke.

Increased values of tau and a decrease of Aß42 were detected in AD in a similar manner as in VaD and acute ischemic events. With special regard to total tau, which is known as an indicator of neuronal damage and discussed to be specifically altered in AD, an increase after stroke was not expected to this degree. In our literature research, we found only limited data dealing with CSF biomarkers after stroke, all of them well in line with results obtained here [2–5]. Tau was described to be significantly increased at day 2–3 (179 %), showing a peak after 1 week (257 %) and after 3 weeks (425 %), normalizing not until 3–5 month (140 %). No significant alteration of p-tau was reported.

For the distinction between VaD and AD, CSF proteins were found to be more altered in AD, such as Aß42 decrease and tau increase, especially phosphorylated forms (Table 2) [6–8]. According to the literature, to differentiate AD from VaD, a combination of all three is recommended [9]. With special regard to AD, a huge amount of data was published. One important aspect is MCI and its progression to AD, which can potentially be predicted with the help of CSF profile [5, 10, 11]. Apparently those MCI patients with a more AD-like CSF profile progress to AD in a shorter period of time, mostly showing low Aß42 and high tau levels [12, 13]. Because CSF biomarkers are already altered very early, there is the possibility to detect AD patients at risk or at very early stages. More precisely, Amyloid-peptide burden and changes in APP metabolism are altered at first up to 10 years before clinical symptoms. Tau proteins are supposed to be rather late markers [14].

The pathogenetic basis for our findings is not clear. CSF Aß42 values are supposed to predict disease progression in AD, but can be altered in VaD, too. Up to now studies have mostly focused either on AD or on stroke and directly comparative analysis is rarely given. Similar to AD, imbalances concerning the total cleavage of APP, with tendencies towards accumulation to amyloid plaques, was reported in VaD. Analogies between cognitive decline in patients after stroke with or without cerebrovascular pathologies prior to this can be seen in comparison to amyloid impact in AD [15].

Total tau might reflect the degree of neuronal damage after ischemia [3, 4]. CSF samples taken immediately after stroke, some days up to several weeks after stroke document an increase of t-tau with a peak after 1 week and a re-normalization 3–5 months later [4]. The level of tau alteration depends on stroke size as well as on time passed since the event [3].

Neuronal apoptosis caused by ischemia is supposed to lead into hyperphosphorylation of tau, and p-tau is not increased directly after stroke [16]. Moreover, as a biomarker it is more useful for AD diagnosis but can be altered in the context of a chronic process like VaD [17]. Whether it can be considered as a new therapeutic target that should be regulated after ischemia and reperfusion process, remains to be determined [18].

Some research has been done using serum in patients after stroke and recently also in AD. Blood samples are of special research interests, because they are much easier to obtain and the analyses can be done sequentially. Increased serum tau was detected in 48 % stroke patients. Those patients with detectable serum tau developed more severe neurological deficits [19–21]. Some other biomarkers were supposed to reflect processes of oxidative stress and inflammation followed by blood–brain barrier dysfunction after cerebral hypoxia. Acute-phase proteins like CRP and NSE were analyzed, too. A larger review concentrated on processes after ischemic brain injury, inflammatory processes contributing to neurodegeneration or by the ischemic event itself [16]. Amyloid balance is supposed to be hampered because of the down-regulation of α-secretase resulting into the non-amyloidogenic pathway of total cleaving of APP, but accumulating soluble neurotoxic amyloid peptides via striking the second pathway using β- and γ-secretases. This explains the finding of AD-like pattern in rodents, starting some days after ischemia—an increase of 200 % of APP in the penumbra on the seventh day post-stroke is described up to 1 year after the event [16]. It also raises the question of the cause of progressive cognitive decline following stroke, inflammatory or degenerative processes.

A striking overlap is found in VaD and AD, such as hypertension, hyperlipidemia, diabetes mellitus and white matter changes subsequently lead into VaD on one hand; on the other hand patients with AD showed more often large vessel abnormalities, like carotid artery stenosis, carotid intimal-medial thinning and bilateral present carotid plaques. Prevalence of cardiovascular risk factors, atrial fibrillation, vessel stiffness and microinfarction increase with age and as result cognitive impairment is not distinctly due to AD or microvascular brain damage alone, rather than a mixture of both [22].

As a drawback of our study the deviation of our control group concerning age has to be mentioned (arithmetic mean 58.9 years). Being around one decade younger than AD or stroke patients with less developed age related changes of the vascular system or degenerative processes, their CSF analysis is expected to show less affected values of the proteins a priori. A control group with more similar age in comparison with the rest would have been more significant; however, since white matter lesions are common in the elderly, such a cohort will be difficult to obtain.

## Conclusions

There is a clear importance to keep in mind the possibility of stroke and vascular degenerative processes, which might lead to increased tau and decreased Aß42 in CSF in a similar manner as in AD. Based on CSF biomarker analysis alone, we could not distinguish stroke from AD patients, since CSF alterations of t-tau, p-tau and Aß42 levels did not differ across groups. Because of its high incidence in elderly population, ischemic events have to be considered in the interpretation of given pathologic CSF profile with increased CSF tau, p-tau and decreased Aß42 levels.

Our conclusions based on our observations reported here are:

1. Ischemic events detected in neuroimaging can cause a pathological CSF profile which resembles those obtained in neurodegenerative dementia/AD
2. A follow-up examination of the biomarkers after several months might be necessary to exclude underlying dementia via normalized CSF profile
3. Patients suffering from stroke show distinct alterations of pathological CSF profile, especially an increase of T-tau
4. In differential diagnosis of cognitive decline after stroke, the pathological biomarker profile can be caused by the ischemic event itself and not by the neurodegeneration alone
